# Case Report: A rare case of oesophageal cellulitis caused by *Klebsiella pneumoniae*: diagnosis, management, and literature review

**DOI:** 10.3389/fmed.2026.1779169

**Published:** 2026-04-17

**Authors:** Jinfeng Yang, Tengyan Wang, Weiwen Yang, Jian Zhu, Chen Pan

**Affiliations:** 1 Department of Gastroenterology, The Affiliated Hospital of Guizhou Medical University, Guiyang, China; 2 Guizhou Provincial Key Laboratory for Digestive System Diseases, The Affiliated Hospital of Guizhou Medical University, Guiyang, China; 3 Department of Gastroenterology, Guizhou Hospital of the First Affiliated Hospital, Sun Yat-sen University, Guiyang, China; 4 Department of Gastroenterology, Affiliated Jinyang Hospital of Guizhou Medical University/The Second People’s Hospital of Guiyang, Guiyang, China; 5 Guizhou Medical University, Guiyang, China

**Keywords:** bacterial oesophageal infection, cellulitis, *K. pneumoniae*, oesophagitis, suppurative oesophagitis

## Abstract

Oesophageal cellulitis caused by *Klebsiella pneumoniae* infection is extremely rare and is often missed because of its atypical symptoms and endoscopic presentation. In this article, a case of a 56-year-old male with poorly controlled diabetes mellitus who presented with chest pain, acid reflux, nausea, vomiting, and weight loss is reported. Imaging revealed progressive, diffuse oedema and thickening of the oesophageal wall, followed by submucosal stratification, fistula formation, and extraluminal gas bubbles. Endoscopic examination revealed progression from erosions in the upper oesophagus to multiple ulcers, fistulae, and intramural abscess formation in the middle and lower oesophagus. Both secretions and tissue cultures confirmed *K. pneumoniae* infection. Following comprehensive treatment, including acid suppression, the administration of effective antibiotics, mucosal repair, nutritional support, and appropriate endoscopic debridement to facilitate drainage, the patient’s symptoms and endoscopic findings significantly improved, and the development of severe complications, such as oesophagotracheal or oesophagomediastinal fistulae, was avoided. This case highlights the need to consider bacterial infection in the differential diagnosis of atypical oesophageal lesions in immunocompromised hosts and emphasises the importance of pathogen-directed, precise anti-infective therapy.

## Introduction

Suppurative oesophagitis is diffuse inflammation of the oesophagus characterised by purulent exudation or abscess formation (1) and is typically caused by bacterial invasion following compromise of the oesophageal mucosal barrier. Acute oesophageal cellulitis is an exceedingly rare, severe and life-threatening form of suppurative oesophagitis in which lesions diffuse through the submucosa and muscular layers (2). It often occurs secondary to foreign body ingestion, iatrogenic injury, or severe reflux. While pyogenic infections can affect any part of the gastrointestinal tract, oesophageal involvement remains uncommon (3–5). Most cases of infection are caused by common pathogens, such as *streptococci* and *staphylococci* (6); infection caused by *Klebsiella pneumoniae* is extremely rare. Conditions associated with immunosuppression, such as diabetes mellitus or malignancy, or long-term use of corticosteroids or immunosuppressants predispose individuals to *K. pneumoniae* infections (7). Although respiratory and urinary tract infections are frequently reported in immunosuppressed individuals, oesophageal involvement, particularly cellulitis, has rarely been documented in the literature. This article reports the successful diagnosis and management of a case of *K. pneumoniae* oesophageal cellulitis in a diabetic patient and discusses its pathogenesis and clinical management strategies with reference to recent literature.

## Case presentation

A 56-year-old Chinese male presented with a two-week history of chest pain accompanied by acid reflux, heartburn, nausea, vomiting, poor appetite, and significant weight loss (approximately 5 kg). He denied having dysphagia, odynophagia, haematemesis, melena, palpitations, chest tightness, cough, sputum, fever, or night sweats. His medical history included a recent diagnosis of diabetes mellitus, which was managed with insulin but with poor glycaemic control. He had a long-standing history of smoking and alcohol consumption. There was no history of corrosive substance ingestion, long-term medication use (including NSAIDs, steroids, or immunosuppressants), or recent consumption of overheated food. Physical examination revealed stable vital signs. Cardiopulmonary examination was unremarkable. Examination of the neck revealed no swelling, tenderness, or subcutaneous emphysema. The abdomen was soft with mild epigastric tenderness and without rebound tenderness or guarding.

Laboratory investigations revealed an elevated white blood cell count (13.5 × 10^9^/L) with an increased neutrophil percentage (86.5%), elevated C-reactive protein concentration (110.85 mg/L), elevated procalcitonin concentration (0.1 ng/mL), and increased erythrocyte sedimentation rate (36 mm/h). Urinalysis revealed glycosuria (3+), proteinuria (1+), and ketonuria (1+). The patient’s haemoglobin concentration was 10. 10%, and his random venous blood glucose concentration was 19.53 mmol/L.

Beta-hydroxybutyrate levels were elevated (2.87 mmol/L). Stool occult blood was positive. Laboratory tests also revealed normal hepatic and renal function, with serum electrolytes within normal ranges. Virological, tuberculosis infection, autoimmune marker, and tumour marker serology tests were negative or showed no significant abnormalities.

**Figure 1:**
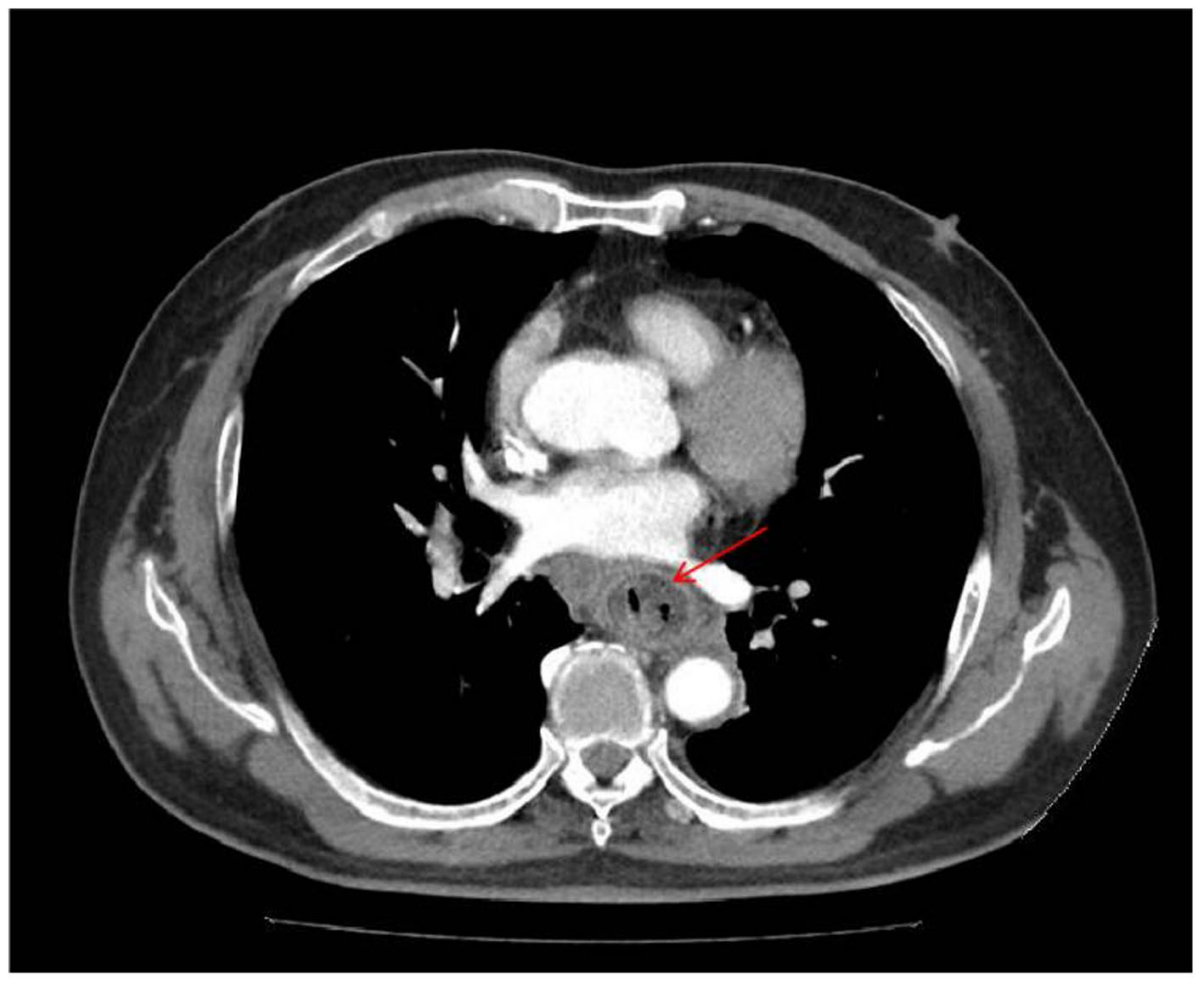
CT showed diffuse thickening with oedema of the oesophageal wall (red arrow indicates the lesion).

**Figure 2:**
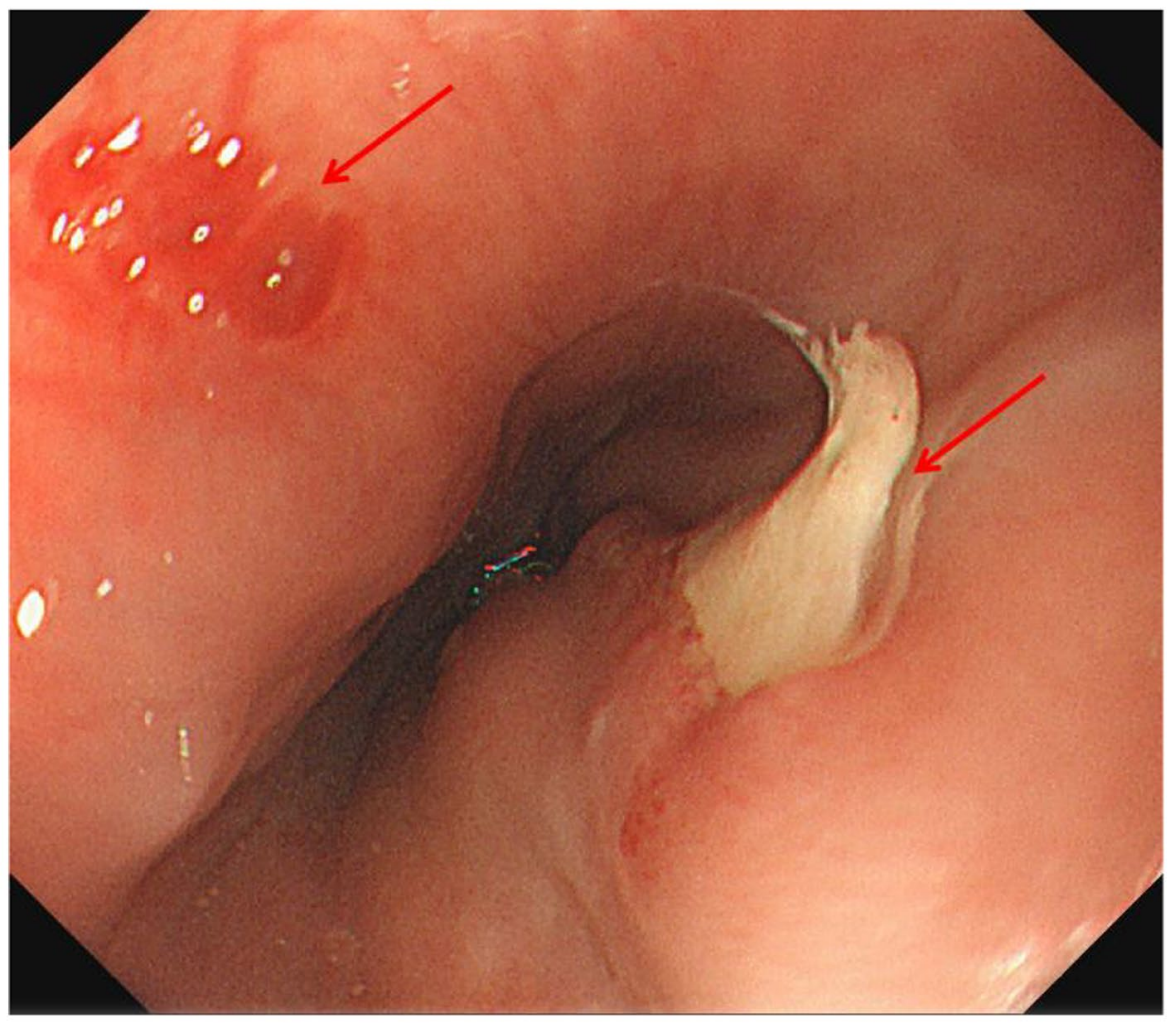
Gastroscopy revealed multiple erosions in the upper part of the oesophagus, with congestion of the mucosa, and submucosal hematoma in the middle part of the oesophagus (red arrow indicates the lesion).

**Figure 3:**
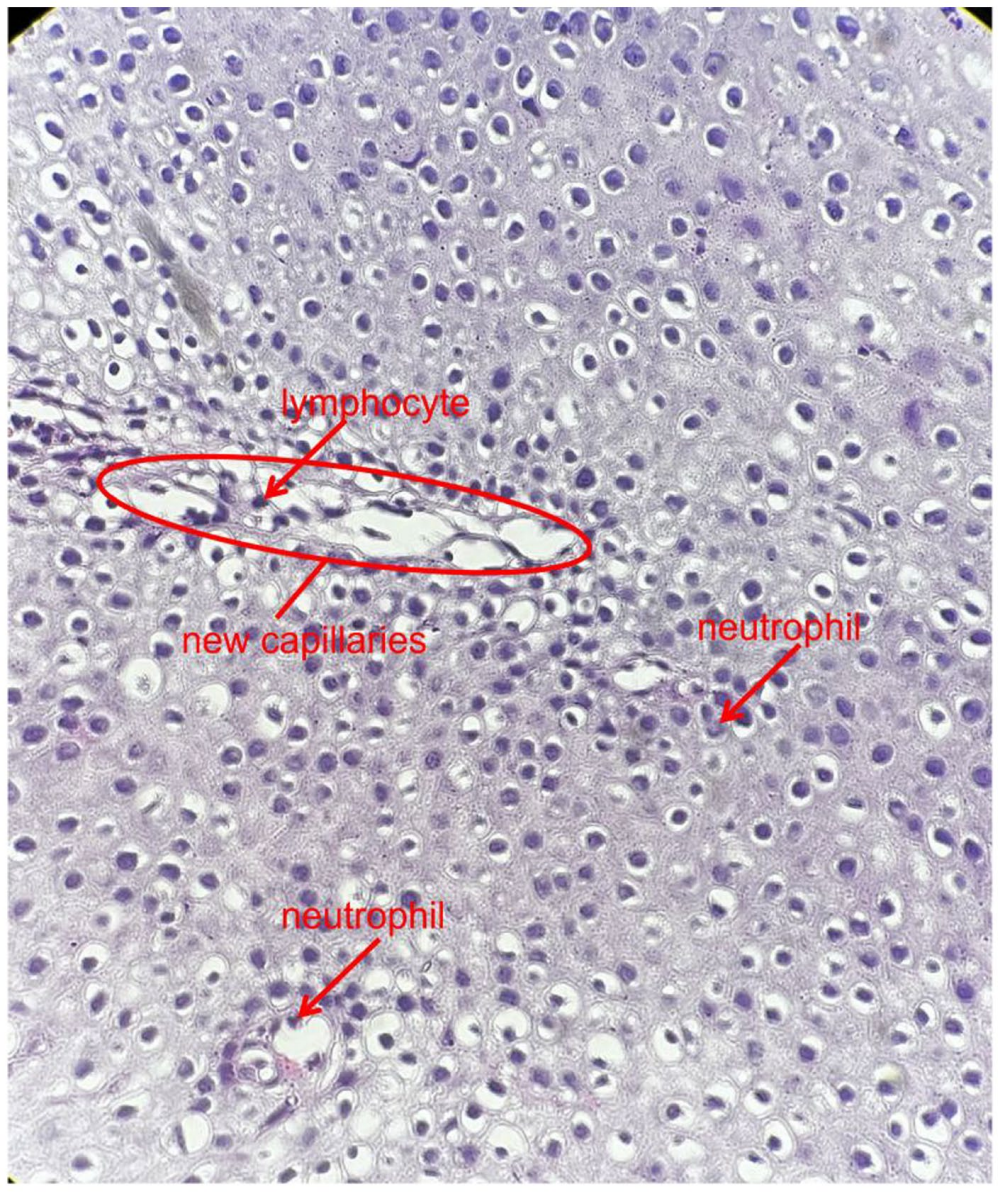
Oesophageal mucosal biopsy showed scattered neutrophils, lymphocytes (indicated by the red arrows) and new capillaries (indicated by the red circles) (H&E, 400×).

Chest CT demonstrated minor inflammation in the lower lobes of both lungs and diffuse thickening with oedema of the oesophageal (Figure 1). Initial gastroscopy revealed multiple erosions in the upper oesophagus and a submucosal haematoma in the mid-oesophagus (Figure 2). Oesophageal mucosal biopsy revealed scattered neutrophils and lymphocytes (Figure 3).

The chronological sequence of clinical events, diagnostic interventions, and therapeutic decisions are summarised in Table 1.

**Table 1:** Clinical timeline: course, diagnostics & interventions.

Time phase	Clinical status	Key diagnostic findings	Core therapeutic interventions	Microbiology
Onset & initial management (days −5 to 0)	Chest pain, heartburn, nausea/vomiting, weight loss	• Lab (Preadmission): Leukocytosis (WBC 13.5 × 10⁹/L), elevated CRP (≥10.85 mg/dL) & PCT (0.10 ng/mL), hyperglycaemia (19.53 mmol/L) with ketosis. • Imaging (CT): Diffuse oesophageal wall thickening and oedema. • Endoscopy #1: Erosions and submucosal haematoma; biopsy showed dense neutrophil infiltration	1. Outside Hospital (Days −5 to −1): • Cefuroxime (1.5 g q12h IV). • Esomeprazole (40 mg bid IV). • L-Glutamine Gluconate Sodium (0.67 g po tid). • Correction of ketoacidosis. 2. On Admission (Day 0): • Symptoms and signs persisted; transferred for further management	Oesophageal specimens sent for culture
Diagnosis & treatment escalation (day +1)	Symptoms persisted	• Lab (Admission): WBC 10.5 × 10⁹/L, CRP 52.62 mg/dL, PCT 0.08 ng/mL; euglycaemia (7.53 mmol/L). • Endoscopy #2: Multiple ulcers with fistula formation. • Imaging (CT): Possible mediastinal involvement	1. Antibiotic: Piperacillin-tazobactam (4.5 g q8h IV) initiated, cefuroxime discontinued. 2. Nutritional Strategy: Fasting initiated; combined parenteral nutrition (including alanyl-glutamine) and enteral nutrition via nasogastric tube. 3. Other: Esomeprazole IV continued; all oral medications stopped	*Klebsiella pneumoniae* isolated. Susceptible to piperacillin-tazobactam and other agents.
Disease progression & intervention (day +5)	No clinical improvement	• Endoscopy #3: Progressing mucosal sloughing	• Intervention: Endoscopic dilation performed. • All systemic and supportive treatments continued.	–
Recovery phase (days +8 to +10)	Symptoms resolved; appetite returned	• Lab: Inflammatory markers normalised. • Endoscopy #4: Significant healing of oesophageal lesions	• Nutrition: Diet advanced to liquid (rice soup, Day +8) then semisolid (rice porridge/purée, Day +10). • Nasogastric tube removed (Day +10). • Oral L-Glutamine Gluconate Sodium (0.67 g tid) resumed	–
Discharge & transition (day +15)	Asymptomatic	All laboratory parameters normalised	• Antibiotic: Piperacillin-tazobactam completed (14-day total course). • Acid suppression: Switched to oral esomeprazole magnesium (20 mg bid) for 8 weeks. • Adjunct: Oral L-Glutamine (0.67 g tid) continued for 8 weeks. • Discharged	–
Long-term follow-up (2 months, 1 year, and 2 years)	• Sustained clinical remission. • Weight gain (~4 kg).	• Asymptomatic; clinical wellness maintained. • Follow-up CT and endoscopy were not performed per patient preference.	All medications discontinued	–

## Diagnosis and initial treatment

**Figure 4:**
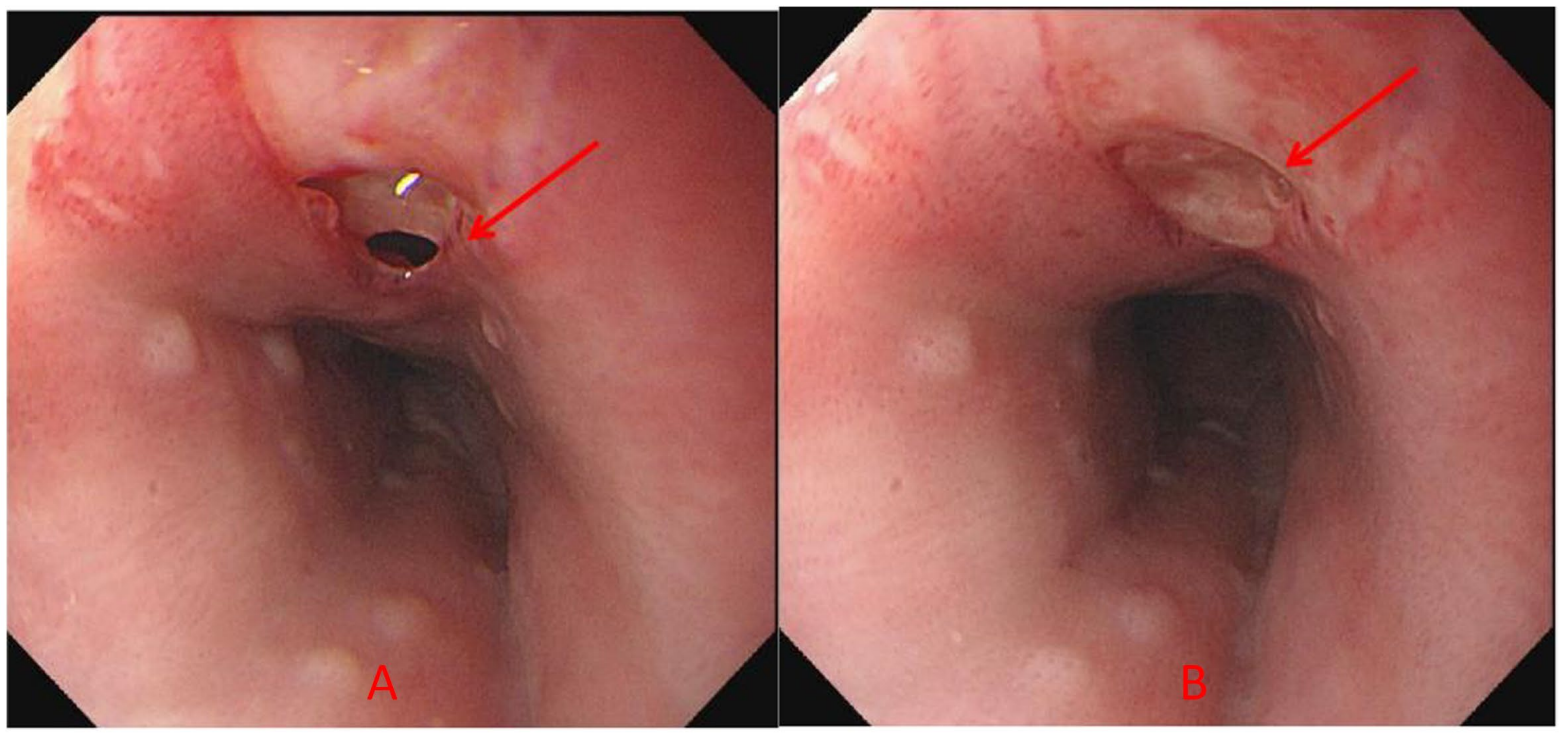
**(A,B)** Gastroscopy revealed multiple fistula openings can be observed 21–30 cm away from the incisors, with diameters ranging from 0.2 to 0.4 cm. When the patient coughs, pus can be seen oozing out, and the surrounding mucosa is congested. On the lateral side of the fistula opening 33 cm away from the incisors, there is a ulcer approximately 0.5 × 0.3 cm in size (red arrow indicates the lesion).

**Figure 5:**
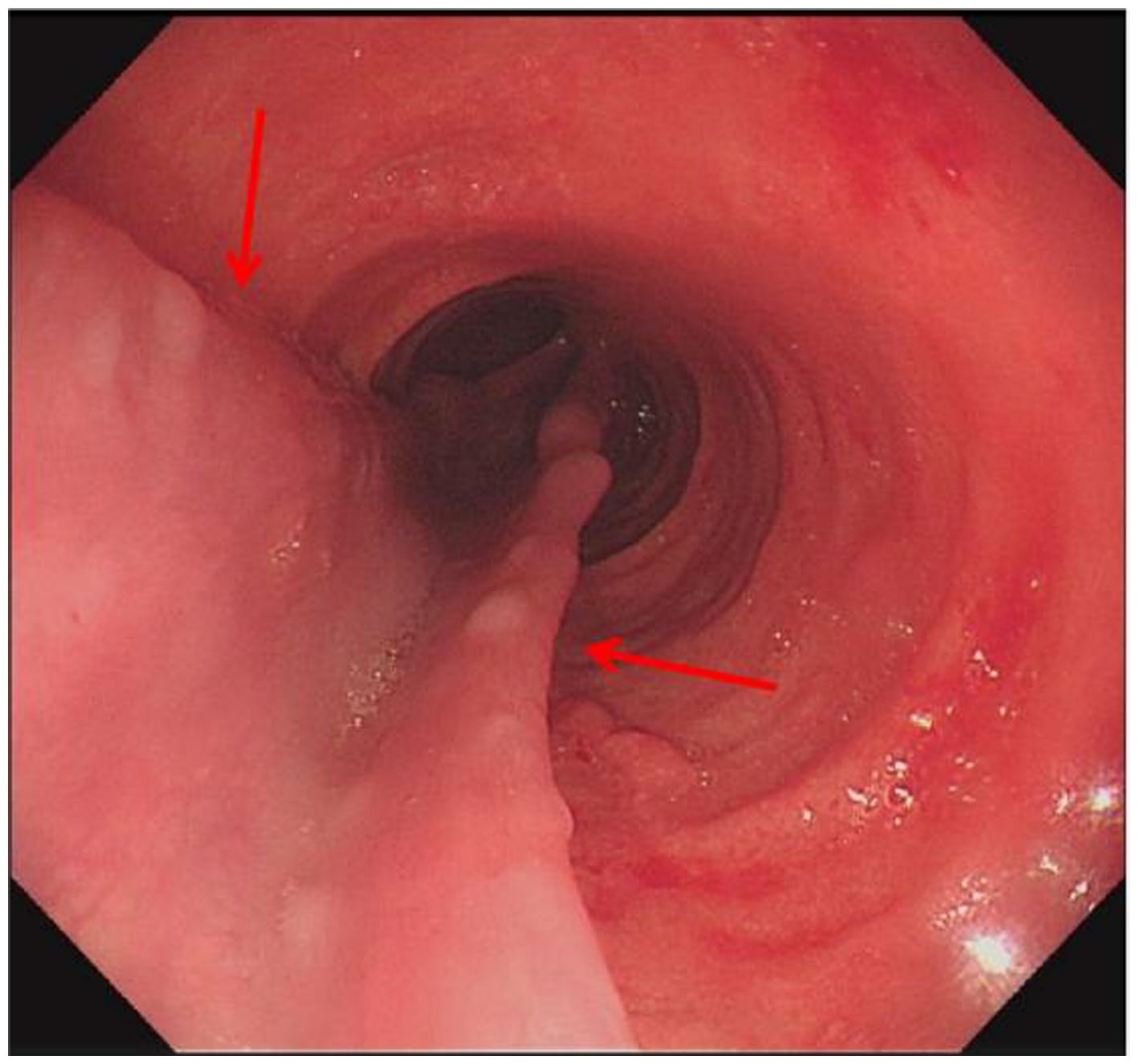
Gastroscopy revealed 21–30 cm away from the incisors, there is a large area of mucosal defect on the left anterior wall of the oesophagus. Below it, there are more granulation tissues and erosions, along with a considerable amount of yellowish-white viscous pus. Biopsy was performed at the base of the granulation tissue, which was brittle and prone to bleeding (red arrow indicates the lesion).

**Figure 6:**
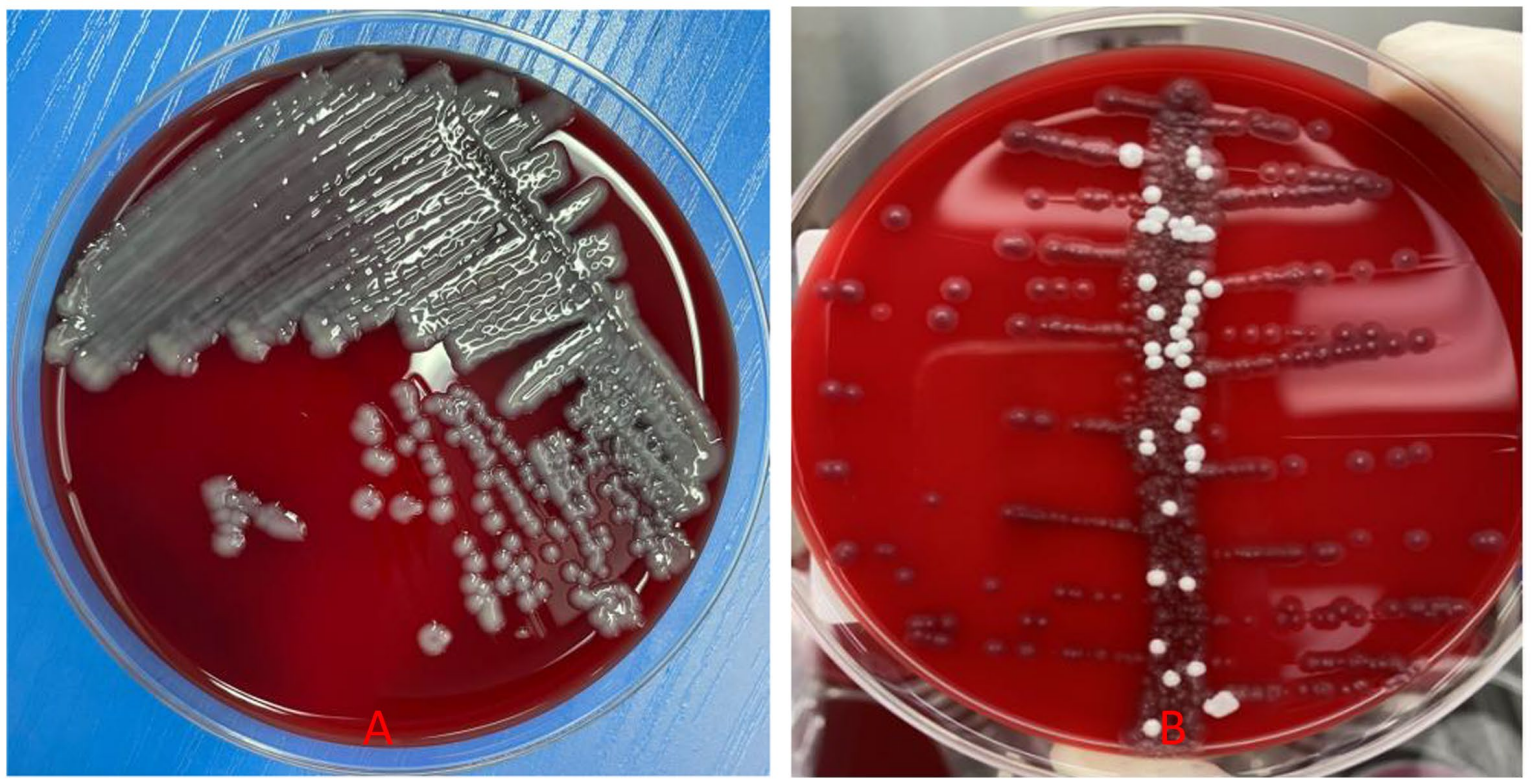
**(A,B)** *K. pneumoniae* was isolated from both the oesophageal fistula secretion and the lesion tissue.

**Figure 7:**
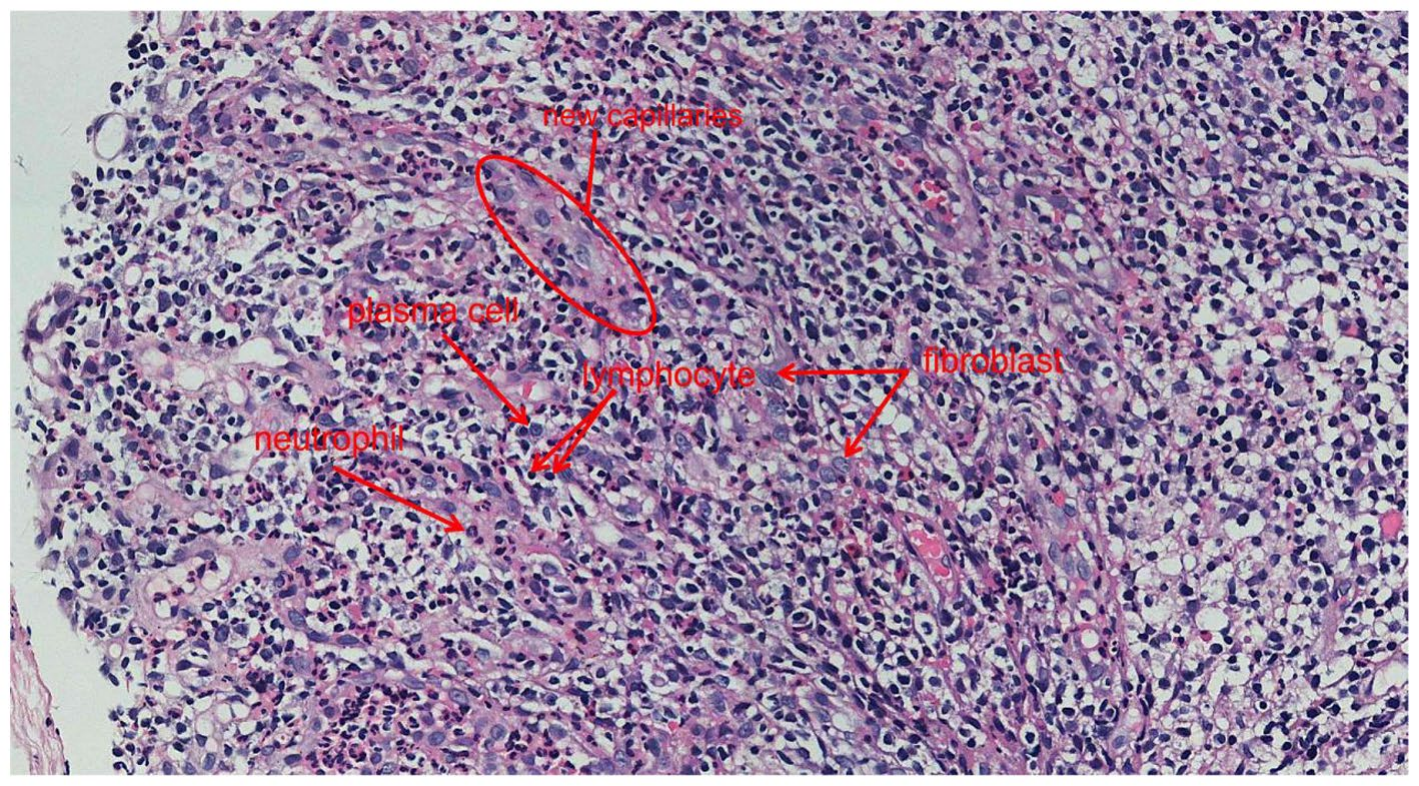
Oesophageal mucosal biopsy showed a large number of new capillaries (indicated by the red circles), neutrophils, and lymphocytes, as well as a large number of fibroblasts (indicated by the red arrows) (H&E, 400×).

**Figure 8:**
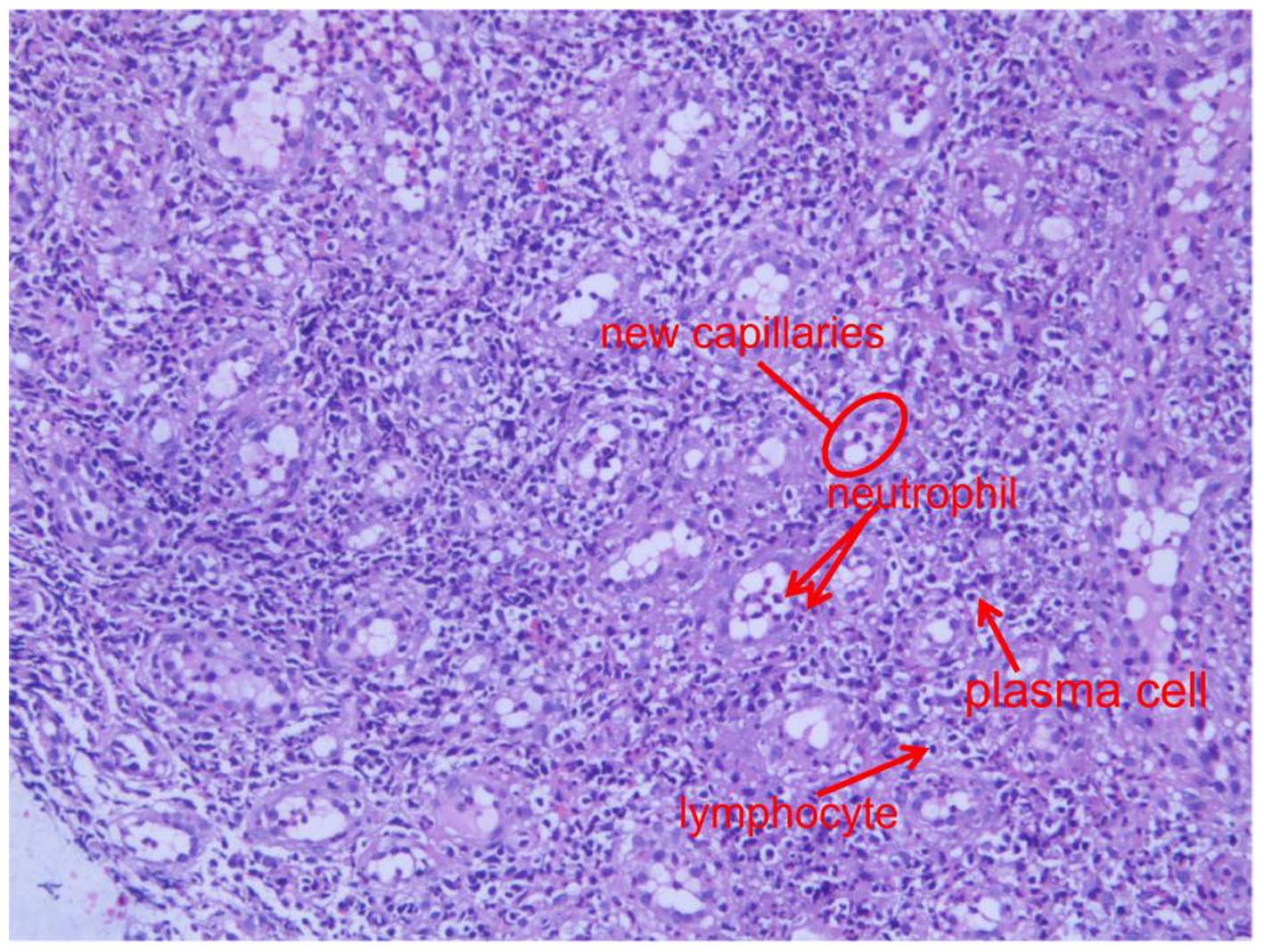
Oesophageal mucosal biopsy showed a large number of new capillaries (indicated by the red circles), neutrophils and lymphocytes, as well as a large number of fibroblasts (indicated by the red arrows) (H&E, 200×).

The diagnosis of acute oesophageal cellulitis was based on the synthesis of the following evidence:

1. *Predisposing Clinical Context:* Poorly controlled diabetes mellitus; subacute chest pain, reflux, weight loss.
2. *Confirmatory Investigations:*
  Laboratory: Laboratory tests revealed leukocytosis, elevated CRP, and procalcitoninemia.
  Imaging: CT revealed diffuse oesophageal wall thickening, intramural gas, and fat stranding — hallmarks of phlegmonous change (Figure 1).
  Endoscopy: Endoscopy revealed progression from erosions to ulcers, fistulae, and mucosal sloughing, indicating a transmural process (Figures 2, 4, 5).
  Microbiology: *K. pneumoniae* was isolated from deep secretions and tissue (Figure 6).
  Histopathology: Biopsy confirmed dense neutrophilic infiltrates, consistent with acute bacterial cellulitis (Figures 3, 7, 8).

Other potential aetiologies, such as oesophageal malignancy, tuberculosis, or viral infection, were excluded through histopathology and serological testing.

**Figure 9:**
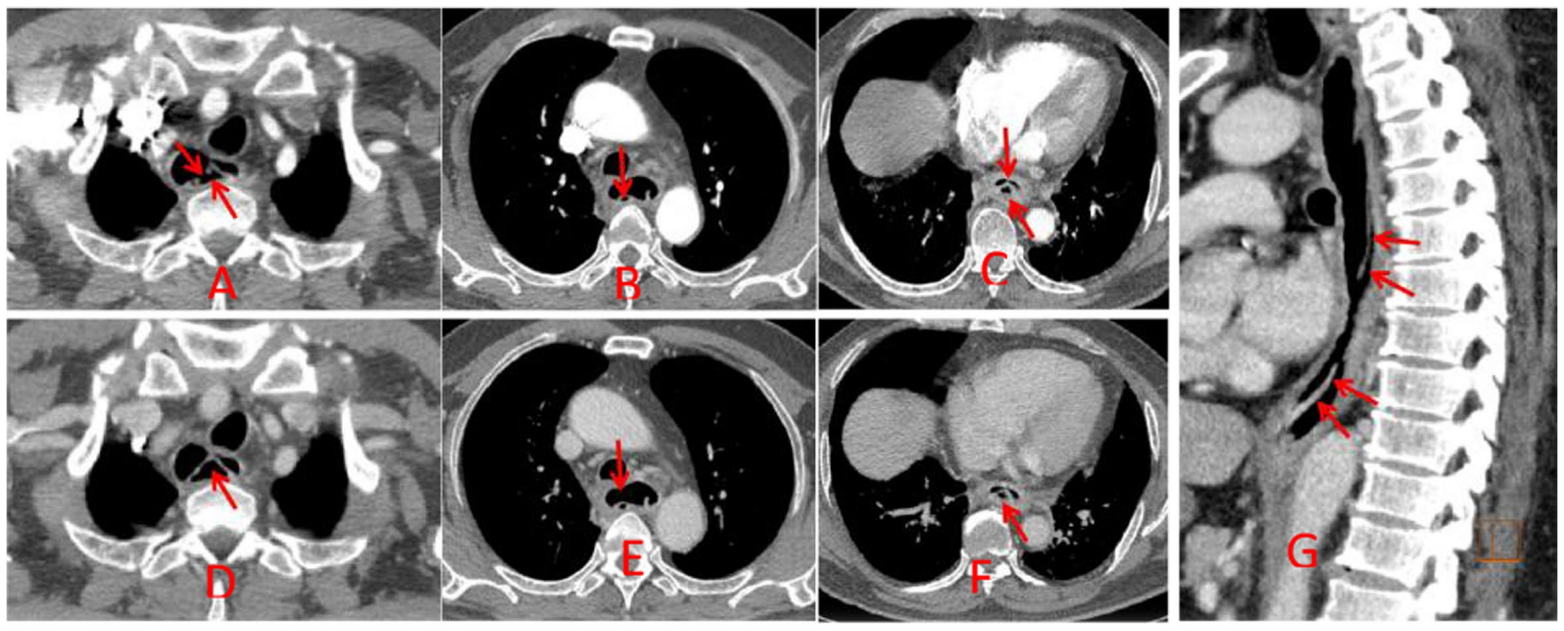
Contrast-enhanced CT in the axial **(A–F)** and sagittal **(G)** planes showed partial discontinuity and thickening of the posterior oesophageal wall (most pronounced in the lower oesophagus). A layered appearance was seen within the gas-containing submucosa. The lesion communicated locally with the oesophageal lumen and showed wall enhancement post-contrast. The peri-oesophageal space was ill-defined with exudative changes.

Consequently, the patient initially received acid suppression, oesophageal mucosal protectants, correction of diabetic ketoacidosis, glycaemic control, and empirical antibiotic therapy (1.5 g of cefuroxime intravenously every 12 h for 5 days). Although inflammatory markers decreased and glycaemic control improved somewhat with this initial treatment, subsequent gastroscopy revealed disease progression, with the development of multiple oesophageal fistulas and ulcers (Figure 4), progressing to mucosal sloughing (Figure 5). Contrast-enhanced CT showed discontinuity of the oesophageal mucosa, intramural gas, and surrounding inflammatory changes, suggesting possible mediastinal involvement (Figure 9). To evaluate for potential perforation suggested by CT, an upper gastrointestinal series with water-soluble contrast was performed under fluoroscopy, which ruled out contrast extravasation and supported continued medical management during surgery. Microbiological and histopathological confirmation was then performed, and oesophageal secretion and tissue cultures revealed *K. pneumoniae* (Figure 6). The bacterial isolate was obtained by culture plate streaking. Antimicrobial susceptibility testing (broth microdilution) revealed that the isolate was susceptible to piperacillin-tazobactam, cephalosporins (including cefuroxime), carbapenems, and fluoroquinolones, with intrinsic resistance to ampicillin. No fungal coinfection was detected. Endoscopic biopsy samples from ulcer margins were fixed in formalin, routinely processed, and stained with haematoxylin and eosin (H&E) for histopathological evaluation. Histopathological examination confirmed severe chronic inflammation and active inflammation (Figure 7).

Despite *in vitro* susceptibility to cefuroxime, clinical and endoscopic progression after 5 days of therapy indicated failure. Given the severity of the infection—which was characterised by transmural inflammation, intramural abscess, and potential mediastinal extension—antimicrobial therapy was escalated to piperacillin-tazobactam (4.5 g intravenously every 8 h for 14 days) because of its broader spectrum, beta-lactamase stability, and superior tissue penetration in complex infections.

## Definitive management and outcomes

**Figure 10:**
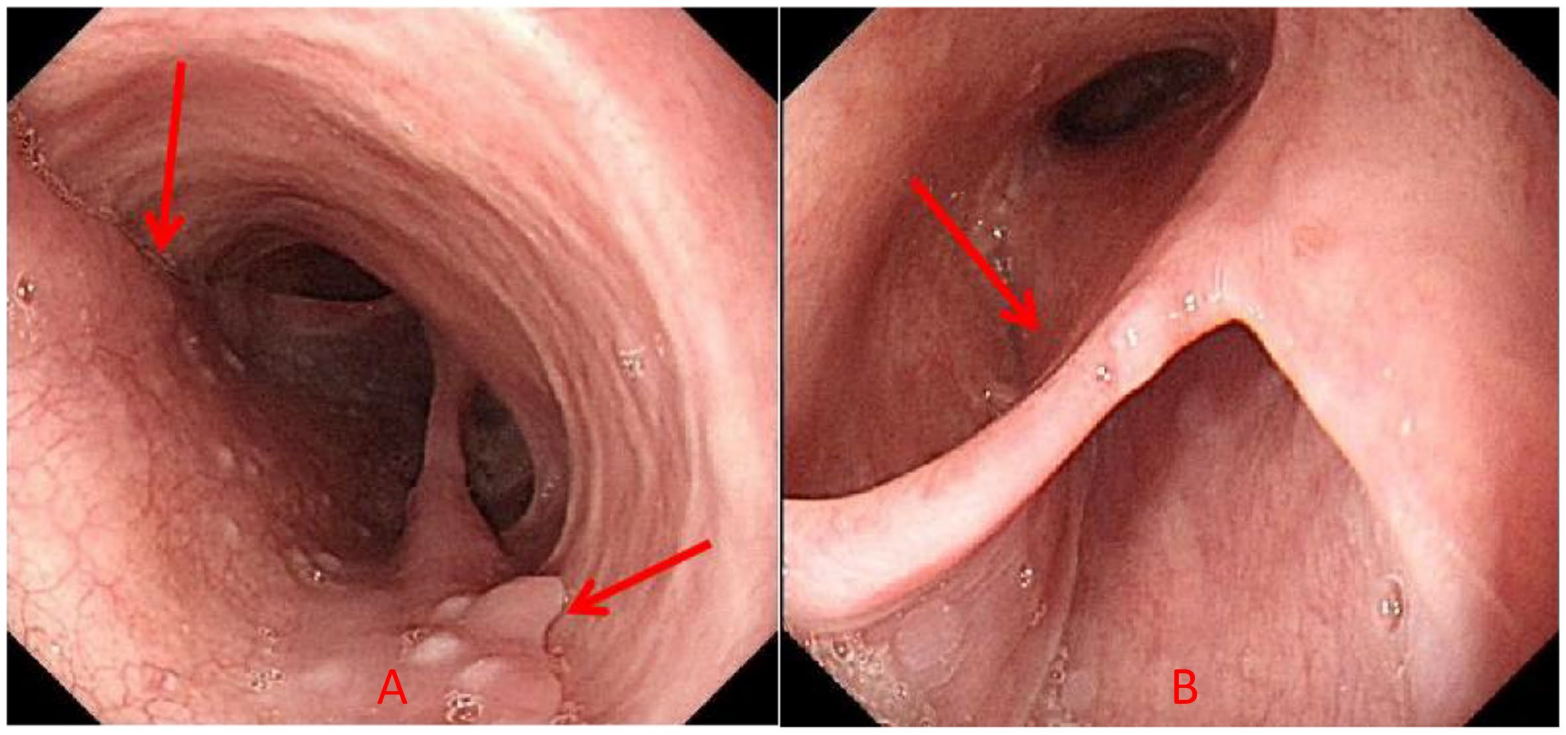
**(A,B)** Gastroscopy revealed that the gastroscopy examination revealed that at a distance of 21–30 cm from the front teeth, a large area of mucosa on the right posterior wall of the oesophagus was stripped, dividing the oesophageal cavity into two parts and forming a mucosal bridge. No pus was attached, and the underlying stripped mucosa was smooth without ulcers. Compared with the previous endoscopy, the mucosal edema had significantly improved (the red arrow indicates the lesion).

On the basis of the patient’s symptoms, endoscopic findings, imaging studies, history of diabetes, and isolation of *K. pneumoniae* from oesophageal fistula secretions and tissue cultures, a diagnosis of *K. pneumoniae*-induced oesophageal cellulitis was established. Antibiotic therapy was escalated on the basis of the antimicrobial susceptibility testing results. The patient was managed with strict fasting, parenteral nutrition, enteral nutrition via a nasogastric tube, acid suppression, mucosal protection, and appropriate endoscopic debridement of abscesses to facilitate drainage. Clinical improvement was observed, with normalisation of the white blood cell count and a reduction in inflammatory markers. Follow-up gastroscopy revealed significant alleviation of the oesophageal lesions (Figure 10), and histology revealed inflammatory cell infiltration and granulation tissue (Figure 8). The patient was discharged with full resolution of his symptoms. Follow-up examinations at 2 months, 1 year, and 2 years post-discharge confirmed sustained clinical remission, although the patient declined repeat endoscopic or imaging evaluation.

## Discussion

Suppurative oesophagitis, a rare but life-threatening condition, typically originates from a compromised mucosal barrier because of factors such as foreign body impaction, iatrogenic injury, or severe gastroesophageal reflux. Common clinical presentations include acute-onset chest pain, odynophagia, and heartburn. However, atypical symptoms such as isolated fever should also be accounted for to avoid diagnostic delays (1, 3). Histopathologically, it is characterised by transmural neutrophilic infiltration, submucosal oedema, and often vascular thrombosis. The microbiological spectrum is predominantly gram-positive, with *streptococci* and *staphylococci* accounting for the majority of cultured cases (6). Against this established backdrop, the present case is remarkable for several reasons.

First, the causative pathogen—*K. pneumoniae*—is exceptionally rare in this context. Its emergence in a patient with poorly controlled diabetes mellitus highlights the critical role of host immunocompromise. Second, the pathogenesis here exemplifies a harmful synergy between a susceptible host and specific bacterial virulence determinants. Diabetic hyperglycaemia impairs neutrophil chemotaxis and phagocytic function, weakening the initial mucosal defence (the “permissive host”). Invading *K. pneumoniae* then deploys key virulence factors: the antiphagocytic polysaccharide capsule evades clearance, lipopolysaccharide (LPS) triggers a potent proinflammatory cascade, and high-affinity siderophore systems (e.g., aerobactin) sequester iron to support rapid proliferation within the tissue niche (8). Critically, components such as LPS are potent inducers of interleukin-8 (IL-8) from epithelial and immune cells (8, 9). The resulting IL-8-mediated neutrophilic recruitment creates a self-amplifying cycle of inflammation: while attempting microbial clearance, intense infiltration directly contributes to tissue necrosis, microabscess formation, and the characteristic progression of phlegmonous cells (10). This detailed mechanistic pathway replaces earlier conjectures and is solidly grounded in contemporary literature. Third, the invasive phenotype (transmural cellulitis, sinus tracts) prompted the consideration of a hypervirulent (hvKP) strain, although genotyping was not performed. HvKP lineages, defined by plasmids carrying genes for enhanced capsule production (rmpA) and siderophore synthesis, are being increasingly reported in community-acquired, metastatic infections beyond the classic liver abscess (10, 11). Our case aligns with this expanding clinical spectrum, underscoring that severe oesophageal presentations may be part of the hvKP disease portfolio. This context heightens the importance of early and precise microbiological diagnosis, especially in an era of increasing carbapenem resistance (10, 11).

The diagnosis of such an atypical presentation hinges on a multimodal synthesis. Formal guidelines are lacking, but a consensus approach integrates (1) characteristic radiological signs, (2) corroborative endoscopic evolution, (3) definitive microbiological evidence, and (4) the exclusion of mimics such as carcinoma or tuberculosis (4, 12–14). This structured framework was applied in the present case. To contextualise the rarity and distinctive features of our case, we present in Table 2 a selection of representative reported cases of acute suppurative oesophagitis for which comprehensive clinical, microbiological, and therapeutic details were available. This focused comparison highlights that, although *K. pneumoniae* is increasingly documented, our case is notable for the definitive isolation of *K. pneumoniae* from deep oesophageal pus and tissue cultures—thereby confirming direct oesophageal invasion rather than secondary bacteraemia or coinfection inferred from isolates obtained solely from blood or pleural fluid.

**Table 2:** Summary of selected reported cases of acute suppurative/phlegmonous oesophagitis.

Author(s), Year	Age (years)/Sex	Predisposing conditions	Predisposing factor(s)	Identified pathogen(s)	Key therapeutic measures	Outcome
Kim et al., 2010 (15)	48/M	Chest pain, dyspnoea, fever	DM (Uncontrolled); Chronic alcoholism; recent minor chest trauma (motorcycle accident)	*Klebsiella pneumoniae* (blood and sputum culture)	1. IV antibiotics (not specified). 2. Surgery: oesophageal myotomies for debridement of submucosal pus. 3. TPN.	Recovered
Karimata et al., 2014 (5)	47/F	Pain, dyspnoea	CRT	Streptococcus milleri (pleural effusion culture)	1. Antibiotics (not specified). 2. TPN.	Recovered
Huang et al., 2017 (7)	60/F	Fever, odynophagia, chest pain	DM (Uncontrolled)	*Klebsiella pneumoniae* and *Pseudomonas aeruginosa* (pleural effusion culture)	1. IV antibiotics: cefoxitin changed to imipenem + gentamycin based on culture results. 2. Surgical interventions. 3. TPN.	Recovered
Shin et al., 2018 (16)	56/M	Chest pain, fever, abdomen	DM; alcoholic liver cirrhosis; hepatitis C virus infection	*Klebsiella pneumoniae* (pleural effusion culture)	1. IV antibiotics: Initial empiric clindamycin + flomoxef. After culture results, changed to piperacillin/tazobactam + ciprofloxacin. 2. Surgery: underwent segmental esophagectomy. 3. TPN.	Recovered
Men et al., 2020 (1)	57/M	Chest pain, fever	Suspected oesophageal mucosal frostbite (due to ingestion of ice water with hail)	*Klebsiella pneumoniae* (blood culture)	1. IV antibiotics: initial piperacillin-sulbactam, then escalated to piperacillin-tazobactam, later changed to levofloxacin based on clinical response (drug fever suspected). 2. Nasogastric (NG) tube feeding. 3. Repeated gastroscopy for irrigation and assessment.	Recovered
Yun et al., 2022 (2)	76/F	Neck pain, foreign body sensation, fever	Suspected mucosal injury caused by a mussel shell fragment in food	Streptococcus Viridans group (sputum culture)	1. IV antibiotics: Initial meropenem + levofloxacin (for pneumonia and oesophagitis). After transfer, switched to ciprofloxacin. 2. Intervention: Endoscopic ultrasonography (EUS)-guided transendoscopic abscess drainage with a hook knife.	Recovered
Lee et al., 2023 (14)	30/M	Case 1: sore throat, odynophagia, fever	DM (Uncontrolled)	*Klebsiella pneumoniae* (blood culture)	1. IV antibiotics (amoxicillin/sulbactam, isepamicin, clindamycin). 2. TPN	Recovered
Lee et al., 2023 (14)	40/M	Case 2: chest pain, melena, haematemesis, fever	DM (Uncontrolled); chronic alcoholism	*K. pneumoniae* (pleural fluid culture)	1. IV antibiotics (ceftriaxone, metronidazole), escalated to piperacillin/tazobactam, thoracic pigtail catheter drainage, later changed to levofloxacin. 2. TPN	Recovered
Lee et al., 2023 (14)	67/F	Chest pain, fever, confusion	DM (Uncontrolled); APN	Culture negative (blood & sputum)	1. IV antibiotics (initial: ceftriaxone & metronidazole; changed to piperacillin/tazobactam; then back to ceftriaxone), supportive care. 2. TPN	Recovered
Gopal et al., 2024 (13)	30/F	Case 2: throat pain, fever, dysphagia	DM (Uncontrolled)	*K. pneumoniae* (throat swab & blood culture)	1. IV antibiotics (piperacillin-tazobactam). 2. Nasogastric (NG) tube feeding.	Recovered
Present case	56/M	Pain, heartburn, ausea/vomiting	DM (Uncontrolled)	*K. pneumoniae* (pus and tissue culture)	1. IV antibiotics (early timely escalated antibiotics) (Cefuroxime was upgraded to Piperacillin-Tazobactam). 2. Endoscopic debridement. 3. TPN. 4. Nasogastric (NG) tube feeding.	Recovered

NP, nothing particular; DM, diabetes mellitus; APN, acute pyelonephritis; CRT, chemoradiotherapy; TPN, total parenteral nutrition.

In this case, contrast-enhanced CT provided pivotal early evidence, demonstrating diffuse oesophageal wall thickening, intramural gas, and perioesophageal fat stranding—the radiologic hallmark of phlegmon (2, 15, 16, 20). Endoscopy vividly revealed progressive destruction, from erosions to ulcers, fistulae, and ultimately mucosal sloughing. This visual timeline, combined with positive cultures from deep tissue, confirmed the diagnosis.

Management of established oesophageal cellulitis demands a dual offensive: source control and robust support. Beyond the prompt administration of effective antibiotics, strict fasting and early nutritional support (enteral/parenteral) are paramount to rest the injured mucosa and mitigate catabolism (1). In this patient, despite initial empirical antibiotic therapy, clinical and endoscopic progression indicated failure. The pivotal decision was to escalate therapy to piperacillin-tazobactam, guided by culture results and, more importantly, by the clinical imperative. This choice is supported by pharmacodynamic principles and guidelines recommending agents with enhanced tissue penetration and beta-lactamase stability for complex, deep-seated infections (17, 18). This case vividly illustrates that *in vivo* clinical response must supersede *in vitro* susceptibility data when directing therapy. The subsequent rapid symptomatic resolution reported by the patient corroborated the objective improvement observed via endoscopy, validating the treatment course. Adjunctive measures, including endoscopic debridement of abscess cavities and meticulous mucosal protection, were integral to success (19). The absence of oesophagomediastinal fistula formation highlights the effectiveness of this early, aggressive, and multifaceted intervention.

### Strengths and limitations

The principal strength of this report lies in the comprehensive longitudinal documentation of a rare disease entity, including serial high-resolution imaging and endoscopic photography that vividly illustrate its progression and resolution under therapy. The diagnosis was fortified by conclusive microbiological and histopathological evidence. However, several limitations must be acknowledged. First, as a single-case report, its generalisability is inherently limited. Second, while the *K. pneumoniae* isolate was thoroughly characterised phenotypically, genotypic analysis to definitively identify hypervirulent (hvKP) or multidrug-resistant markers was not performed. Such data would have provided deeper insights into the pathogenicity of the strain. Finally, the patient declined long-term endoscopic follow-up, precluding a definitive anatomical assessment of healing despite sustained clinical remission.

### Clinical implications and future directions

This case extends beyond a singular rare event, offering broader insights for clinical practise and research. First, the aetiology of severe oesophagitis in immunocompromised hosts may include atypical, aggressive pathogens such as *K. pneumoniae*. This necessitates a shift in diagnostic mindset, especially in patients with poorly controlled diabetes, prompting earlier consideration of such organisms even in the absence of classical mechanical triggers.

Second, the clinical course underscores a critical therapeutic principle: in deep-seated infections within compromised hosts, antimicrobial therapy must be dynamically guided. Reliance on empirical regimens active against typical flora (e.g., streptococci) may prove inadequate. The pivotal decision to escalate to piperacillin-tazobactam, driven by clinical progression despite initial therapy, highlights that *in vivo* response must often supersede *in vitro* susceptibility patterns in the management of such severe, tissue-invasive infections.

Finally, while genotypic confirmation was unavailable, the invasive phenotype observed aligns with concerns regarding hypervirulent *K. pneumoniae* (hvKP) strains and their expanding disease spectrum. This potential association reinforces the importance of striving for precise microbiological characterisation in similar cases, as it influences prognosis and infection control. Future collaborative studies are needed to define the true prevalence, risk factors, and optimal management strategies for such rare but life-threatening oesophageal infections, potentially informing future guideline development.

## Conclusion

In conclusion, this case establishes *K. pneumoniae* as a rare but critical pathogen in the spectrum of suppurative oesophagitis, particularly in diabetic hosts. This highlights the necessity of incorporating aggressive gram-negative bacteria into the differential diagnosis for atypical, severe oesophageal presentations in immunocompromised individuals. Successful management hinges on a multimodal approach: early recognition through advanced imaging and endoscopy, prompt and decisive escalation of antimicrobial therapy guided by both microbiology and clinical response, and rigorous supportive care to facilitate mucosal healing and prevent catastrophic complications.

## Data Availability

The original contributions presented in the study are included in the article/supplementary material, further inquiries can be directed to the corresponding authors.
